# Changes in the ultrasound presentation of hepatocellular carcinoma: a center’s three decades of experience

**DOI:** 10.1007/s40477-024-00888-7

**Published:** 2024-04-07

**Authors:** Lydia Giannitrapani, Simona Amodeo, Luigi Mirarchi, Antonino Terranova, Aurelio Seidita, Chiara Mozzini, Daniela Cabibi, Giuseppe Brancatelli, Anna Licata, Maurizio Soresi

**Affiliations:** 1 Department of Health Promotion Sciences, Maternal and Infant Care, Internal Medicine and Medical Specialties (PROMISE), University of Palermo, Palermo, Italy; 2 Department of Medicine, ASST Mantova, C. Poma Hospital, Mantua, Italy; 3 Department of Biomedicine, Neuroscience and Advanced Diagnostic (Bi.N.D.) Section of Radiological Sciences, University of Palermo, Palermo, Italy; 4 Institute for Biomedical Research and Innovation (IRIB), National Research Council, Palermo, Italy

**Keywords:** HCC, Liver cirrhosis, Ultrasound, Echo-pattern, HCV, NAFLD

## Abstract

**Purpose:**

Ultrasound (US) surveillance is a cornerstone for early diagnosis of HCC, anyway US presentation has undergone significant changes. With the aim of evaluating the effects of US surveillance program in the real-world clinical practice, we wanted to evaluate US presentation of HCCs over the last 30 years and the differences of HCCs presentation according to etiology.

**Methods:**

174 patients diagnosed between 1993 and 98 (G1), 96 between 2003 and 08 (G2), 102 between 2013 and 18 (G3), were compared. US patterns were: single, multiple or diffuse nodules. The echo-patterns: iso-, hypo-, hyper-echoic, or mixed.

In G1, the HCC diagnosis was mainly histologic; in G2 by EASL 2001 and AASLD 2005, in G3 AASLD 2011, EASL 2012, and AISF 2013 guidelines.

**Results:**

HCV was the most frequent etiology, dropping between G1 (81%) and G3 (66%) (*P* < 0.01), metabolic increased between G1 (5%) and G3 (14%) (*P* < 0.01). Single HCC was more prevalent in G3 vs G1 (65.6% vs 40%) (*P* < 0.0001), multiple nodules in G1 (50%) vs G3 (33.3%) (*P* < 0.02) and diffuse in G1 (16%) vs G2 (2%) and vs G3 (1%) (*P* < 0.001). The most frequent echo-pattern was hypo-echoic G1 (50%) vs G2 (79%) and G1 vs G3 (65%) (*P* < 0.01). Iso-echoic pattern was the least frequent (7–12%). Mixed pattern decreased from G1 (28%) to G3 (12%) (*P* < 0.002). In G3 there were more multiple or diffuse HCCs in metabolic (*P* < 0.03).

**Conclusion:**

US presentation became less severe due to surveillance programs. HCV remains the most frequent cause, an increase in metabolic etiology has been shown throughout the decades.

**Graphical abstract:**

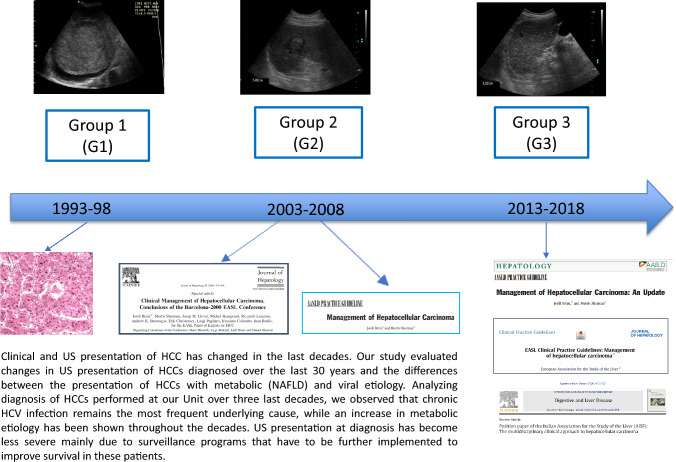

## Introduction

HCC is the most frequent primary liver cancer and with approximately 854,000 new cases per year [1] is the second leading cause of cancer-related death globally [2].

The geographical distribution of HCC is heterogeneous and closely related to the distribution of its risk factors. It is highly incident in East Asia and Sub-Saharan Africa with more than 85% of all cases [3]. In Europe the incidence is lower, with the exception of the south where it is higher [4]. In Italy, 12,800 new cases of HCC were diagnosed in 2018, about 3% of all new cases of cancer (AIRTUM register, 2018). It has a severe prognosis, with a 5-year survival of 20%, without appreciable differences across the national territory [5].

In 90% of cases, HCC develops in a cirrhotic liver [6], which is why the risk factors for both pathologies are coincident. The main causes of LC on which HCC can arise are chronic liver disease caused by HBV, HCV, alcohol abuse, exposure to aflatoxin, and hereditary metabolic pathologies (α1 anti-trypsin deficiency, hemochromatosis, Wilson disease, porphyria). In recent years data in the literature have shown a reduction in HCC cases related to viral infections and an increasing number arising in the context of the metabolic syndrome [7, 8]. This trend is probably related to the vaccination campaign against HBV, the introduction of antiviral treatments for HCV and HBV, and surveillance programs for patients with LC. With the increased prevalence of metabolic syndrome, it is foreseeable that in the coming years this will become the predominant cause of HCC, exceeding infection-based cases at least in the Western world [9].

These epidemiological changes, improvements in imaging techniques, and twice-yearly US surveillance programs for at-risk populations are changing the clinical presentation and the prognosis of HCC. Many evidences have proven that screening program performing US as HCC surveillance in chronic liver disease are effective in increasing curative treatments of HCC and in reducing mortality and that US surveillance improves the prognosis of patients at high-risk for HCC development [10].

The aim of this study was to analyze, in terms of number of lesions and their echo-patterns, changes in the US presentation of HCCs diagnosed at our center between 2013 and 2018, comparing them with those observed in two previous periods, 1993–1998 and 2003–2008 and to evaluate differences in US presentation between the forms of HCC related to NAFLD and viral etiology, with the possible clinical implication of supporting and implementing ultrasound surveillance in cirrhotic patients.

## Materials and methods

### Patients

Our case study was divided into three groups based on the HCC diagnosis period. G1 included 174 patients (125 M/49 F) with a diagnosis of HCC made between 1993 and 1998, G2 included 96 patients (56 M/40 F) with HCC diagnosed between 2003 and 2008, and G3 consisted of 102 patients (73 M/29 F) with HCC diagnosed between 2013 and 2018. In the first two groups, all HCCs developed on LC. In the third period, we found 2 cases of HCC without underlying LC. Age, gender, serum markers for hepatitis B and C viruses, anti HDV (in HBV positive subjects), alcohol consumption, serum AFP levels and the main liver function parameters were recorded for all patients at the time of HCC diagnosis. In the absence of viral or alcoholic liver disease, ANA, ASMA, anti-LKM1, AMA, serum ferritin and iron were measured. Patients were classified as viral and non-viral according to etiology. The serum anti-HCV test was performed using a 2nd and 3rd generation Enzyme Immuno-Absorbent Assay (ELISA, Ortho Diagnostic Systems, Raritan, New Jersey) for G1 and G2-G3 patients respectively, according to the manufacturer's instructions. Positive anti-HCV samples were confirmed using a 2nd generation anti-HCV recombinant immunoblot assay (RIBA II, Chiron Corporation, Emeryville, California) for G1, and viral RNA analysis by PCR for G2 and G3. HBV serological markers were tested using commercially available kits (Abbott Laboratories, Chicago, IL, USA) for HBsAg research. If tests for both HBsAg and anti-HCV were negative, a diagnosis of non-viral etiology was made. Patients with a non-viral etiology were divided into:

- Alcoholic: if the daily intake of ethanol was > 40 g for women and > 30 g for men, for more than 5 years, in the absence of other causes of liver damage.
-Metabolic (post-NASH): if patients were negative for HBsAg or anti-HCV antibodies, there was no known history of alcohol abuse, the diagnosis of autoimmune liver disease or genetic liver disease was excluded, and if they had a positive medical history for metabolic syndrome.
-Other etiologies: hemochromatosis, Wilson disease, primary biliary cholangitis, primary sclerosing cholangitis, and alpha-1 antitrypsin deficiency.

This study was reviewed and approved by the Ethics Committee of the “Policlinico P. Giaccone” Palermo University Hospital, in the session N°11/2021 of the 15/12/2021.

### HCC diagnosis

In G1, the diagnosis of HCC was histologic in 26.6%, by liver biopsy (39/174) or surgical specimen (8 cases); in the rest of the cases, it was based on multiple concordant imaging techniques (US, baseline and post-lipiodol administration computed tomography, selective angiography) and with serum AFP levels > 200 ng/ml [11]. In G2, the criteria of the EASL 2001 and AASLD 2005 guidelines were used for the majority of the diagnoses [12, 13] and in 29 patients (30%) it was made by histology. In G3, 9.2% of HCC diagnoses were histological, while in the remaining cases they were formulated according to the AASLD 2011, EASL 2012, and AISF 2013 guidelines [14–16]. US presentation was classified in all groups according to Otho as Single, Multiple, or Diffuse [17].

### LC diagnosis

The diagnosis of a possible underlying LC, in all three periods, was based on histological findings or unequivocal clinical and biochemical signs associated with at least one positive imaging technique (US or computed tomography). Furthermore, in the third group, Elastography (Fibroscan Echosens) was performed on 27% of the patients. LC was staged according to the Child–Pugh's score [18].

### US

US of the upper abdomen was performed in the morning after at least 8 h fasting, using a Toshiba SSA 270 A real time device with a 3.5 MHz convex probe for G1, a Philips 5000 real-time device HDI with convex probe with a 5–2 MHz multi-frequency system for G2 patients and a Philips IU 22 US system. A 1–5 MHz convex transducer for G3 were used.

All lesions were examined also by color and power Doppler ultrasound. The liver was examined in inter- and/or subcostal planes with a fan-like motion allowing assessment of both the hepatic parenchyma and the intrahepatic bile ducts. Number of focal liver lesions (solitary, multiple), the respective ultrasound characteristics of the focus and size of the tumor (maximum diameter) were evaluated. Grey scale echogenicity of the focal lesions was classified in comparison with the adjacent liver parenchyma. We distinguished hypoechoic, hyperechoic, and isoechoic lesions; mixed pattern was characterized by the coexistence of two the previous echo structures in the same lesion.

### Statistical analysis

Data are expressed as mean ± standard deviation; the differences between the means and frequencies between the two groups were calculated with Student’s *t*, *χ*2, Fisher's exact test; when the distribution was not normal, data were expressed as median and interquartile range (IQR) and their difference evaluated with Mann Whitney test; Spearman rank correlations were used when appropriate. The results were considered significant if *P* < 0.05.

## Results

The diagnosis of HCC had been made during twice-yearly US surveillance only in 20/174 (11.5%) in G1; this was lower than in G2, where the patients under surveillance were 64/96 (66%) (*P* < 0.0001), and G3, where they were 78/102 (76.4%) (*P* < 0.0001). Between G2 and G3 there were no statistically significant differences (*P* = ns).

Table 1 shows demographic and biochemical data on the three groups. The mean age increased progressively, and statistically significantly, from G1 to G3: in G1 it was 65 ± 9 years, in G2 69 ± 8 years, in G3 72 ± 11 years (*ρ* = 0.4; *P* < 0.0001). This significant increase was also confirmed for patients with HCV etiology (*ρ* = 0.35; *P* < 0.005). There was no statistically significant age difference for HBV, even though there was a progressive but not significant increase in age in patients with non-viral etiology (*P* = ns).

**Table 1:** Patients of the three groups divided according to aetiology, age, and sex

	M/F (%)			*P* <	Age			*P* <
	G 1	G 2	G 3		G 1	G 2	G 3	
Total	125/49	56/40	73/29	ns	65 ± 9	69 ± 8	72 ± 11	0.0001^a^
HCV	97/43 (81)	40/35 (78)	43/25 (66)	ns	65 ± 8	71 ± 7	73 ± 10	0.001^b^
HBV	10/2 (7)	7/1 (8)	9/1 (10)	ns	58 ± 7	56 ± 11	55 ± 18	ns
HBV/HCV	4/0 (4)	2/1 (3)	2/0 (2)	ns	60 ± 8	71 ± 3	68 ± 10	ns
Alcohol	8/1 (6)	3/0 (2)	5/0 (5)	ns	67 ± 8	71 ± 5	68 ± 8	ns
Metabolic	4/2 (5)	3/2 (5)	11/3 (14)	ns	69 ± 7	69 ± 13	72 ± 10	ns
Mixed	1/1 (1)	1/0 (1)	2/0 (2)	ns	64 ± 6	–	66 ± 7	ns
Other	1/0 (4)	2/1 (2)	1/0 (1)	ns	63 ± 9	69 ± 6	–	ns

Age in the three groups ^a^*ρ* = 0.4; ^b^*ρ* = 0.35

**Fig. 1:**
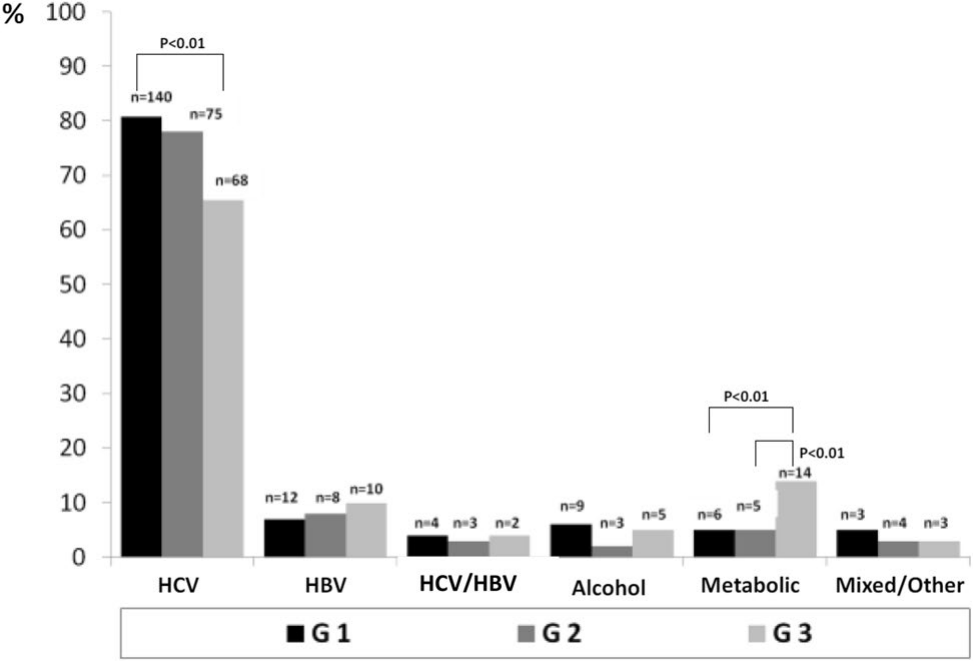
HCC etiology in the three study groups

Figure 1 shows the etiology of HCCs in the three study groups. In all three groups, the most frequent etiology was HCV, but its frequency decreased in G3 where it was significantly lower than in G1: 68/102 (66%) vs 140/174 (81%) (*P* < 0.02). There were no significant differences between G1 vs G2, 75/96 (78%, *P* = ns) or in G2 vs G3 (*P* = ns). On the contrary, there was a progressive, increasing trend in the number of HCCs with metabolic etiology from G1 (4%) to G2 (5%) and to G3 (14%), with a statistically significant difference between G1 vs G3 (6/174 vs 14/102; *P* < 0.002) as well as G2 vs G3 (5/96 vs 14/102; *P* < 0.05). HBV infection alone or HCV/HBV co-infection showed no statistically significant differences.

**Fig. 2:**
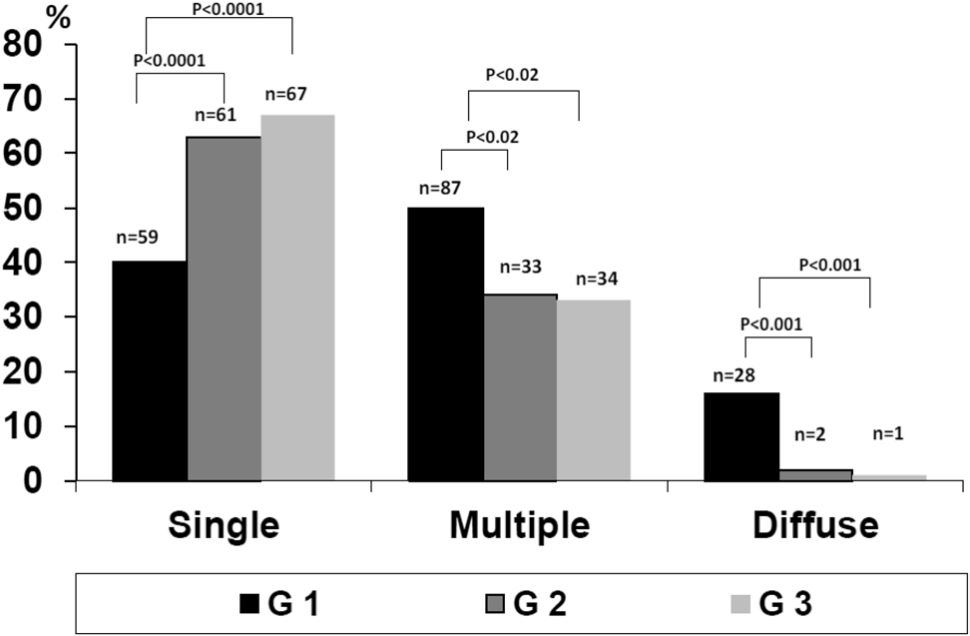
US presentation in the three groups according to Otho’s classification

Figure 2 shows the US presentation according to Otho’s classification of HCC echo-patterns at the time of diagnosis in the three study groups. The percentage of patients with single HCC progressively increased, and it was significantly higher in G3 (65.6%) vs G1 (40%) (*P* < 0.0001) and in G2 (63.5%) vs G1 (40%) (*P* < 0.0001), whereas between G2 and G3 there was no statistical difference (*P* = ns).

Conversely, the frequency of multiple HCCs in G1 (50%) was statistically and significantly higher than in G2 (34.4%) and G3 (33.3%) (both *P* < 0.02), while no difference was found in the comparison between G2 and G3 (*P* = ns). Similarly, diffuse HCC was more frequent in G1 (16%) than G2 (2%) or G3 (1%), (both *P* < 0.001), though no significant difference was found between G2 and G3 (*P* = ns).

Table 2 shows results about size (considering the largest nodule in multifocal patterns) and the significant differences in the 3 groups. Both in the presentation as a single or multiple nodules, there was a statistically significant reduction in size between G1 and G2, and G1 G3 (*P* < 0.0001) but not between G2 and G3.

**Table 2:** Differences in the size of HCC in the three groups

	Single			
	G1 *n* = 59	G2 *n* = 61	G3 *n* = 67	*P* <
Median size (IQR)	4.1 (3.1–5)	2.3 (1.7–3)	2.1 (1.5–3)	G1 vs G2 0.0001 G1 vs G3 0.0001 G2 vs G3 ns

	Multiple			
	G1 *n* = 87	G2 *n* = 33	G3 *n* = 34	*P* <
Median size (IQR)	5 (3.2–7)	2.6 (1.6–3.0)	2.0 (1.5–2.9)	G1 vs G2 0.0001 G1 vs G3 0.0001 G2 vs G3 ns

**Fig. 3:**
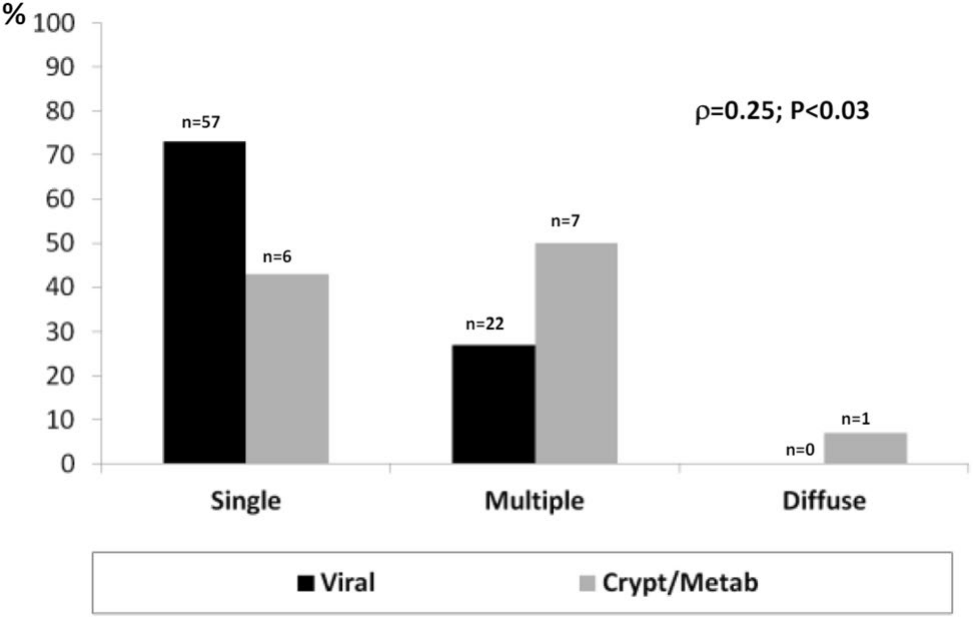
US presentation in viral vs metabolic HCCs according to Otho’s classification

In G3, comparison of Otho’s US presentation of HCCs with viral and metabolic etiology revealed that, in the latter, there was a higher frequency of multiple or diffuse HCCs (*ρ* = 0.25, *P* < 0.03) (Fig. 3). LC was present in 13/14 of patients with metabolic vs 78/79 of viral etiology (*P* = ns). A twice-yearly US surveillance program was performed in 5/14 (35%) of metabolic vs 62/79 (78.4%) of viral LC patients (*P* < 0.0001) (Fig. 3).

**Fig. 4:**
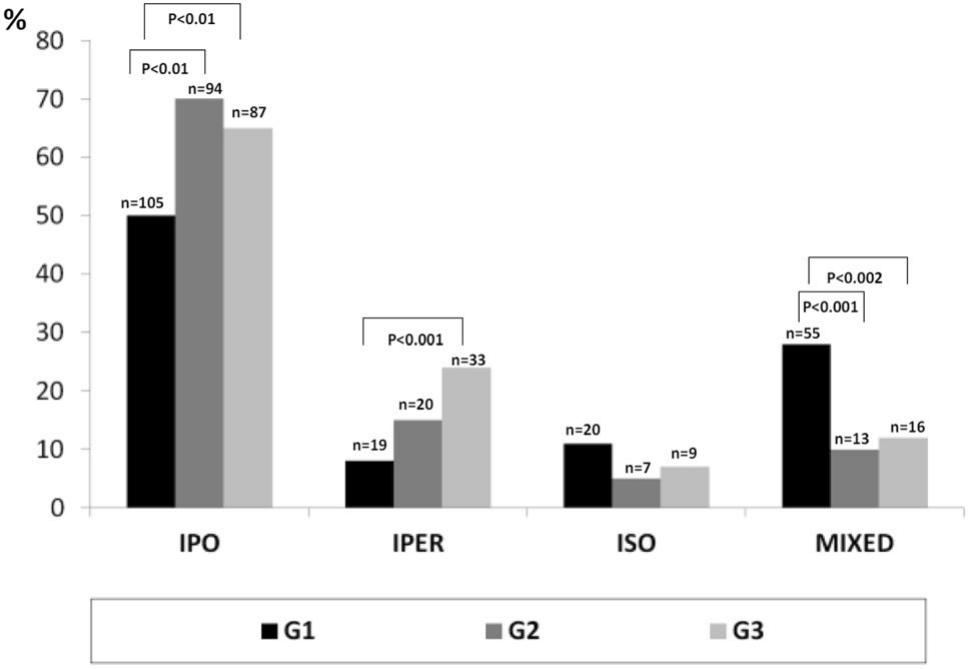
Echo-patterns in the three study groups

Figure 4 shows the echo-pattern of HCC nodules in the three periods. The most frequent was the hypo-echoic (50–70%), which increased significantly in the second and third periods compared to the first: G1 (50%) vs G2 (70%) (*P* < 0.001) and G1 vs G3 (50% vs 65%; *P* < 0.001); between G2 and G3 the frequency overlapped. The hyper-echoic pattern showed the same behavior, becoming significantly more frequent in the third period when compared to the first one (24% vs 8%; *P* < 0.0001). The iso-echoic structure was the least frequent (7–11%) and did not show significant changes over the three periods. The mixed pattern gradually decreased significantly between G1 and G2 (28% vs 10%) (*P* < 0.001) and between G1 and G3 (28% vs 12%) (*P* < 0.002).

## Discussion

Our results, in accordance with recent literature data [11], confirm that over the past few decades the etiology as well as the clinical and US presentation of HCC have undergone changes.

Our previous study had underlined the features of the changing epidemiology of HCC regarding clinicopathologic data including Child–Pugh and BCLC staging [8].

As regards our study of disease severity at diagnosis according to Otho’s classification, the US presentation of HCC at the time of diagnosis was less severe in G3 and G2 than in G1. In fact, it is evident that the frequency of patients with single HCCs at diagnosis was progressively and significantly higher in G3 compared to the other two groups and the presentation of multiple or diffuse HCCs gradually decreased from G1 to G3. In G2 and G3 patients, the frequency of twice-yearly US was significantly higher, and this, alongside improvements in US technology, allows us to affirm that the US surveillance programs already proposed by the EASL 2001 guidelines [19], despite their limitations in the diagnosis of early and very early cancer, have allowed for the earlier diagnosis of HCC and therefore better prognosis.

An interesting finding emerges from the comparison of the presentation of HCCs with viral vs metabolic etiology. We restricted this comparison to the 2013–18 period only because in the previous two periods the prevalence of metabolic HCC was limited. What we observed is that HCCs with metabolic etiology showed more severe staging according to Otho than viral (*ρ* = 0.4 *P* < 0.0001). One possible explanation is the reduced percentage of patients undergoing surveillance; in our study only 15.4% of metabolic patients underwent bi-annual screening vs 77.9% of those with viral etiology. Patients with cryptogenetic/metabolic chronic liver disease, in fact, are often not aware of having a liver disease and do not undergo periodic checks; therefore, finding a neoplasm in a more severe stage may depend on the lack of surveillance [20].

The findings from our study, in agreement with the increasing prevalence of metabolic forms, open a discussion on the need to expand US screening for HCC criteria. In fact, AASLD, EASL, APASL, and AISF guidelines recommend that patients with LC undergo US surveillance for HCC every 6 months [21] but in NAFLD/NASH patients, this suggestion is controversial. Many data from the literature besides the present study suggest that in NASH HCC may also occur on a non-cirrhotic liver [22] but caution is needed in expanding the screening to all patients with US evidence of liver steatosis. In fact, the limits of this method are well-known, especially in obese patients. Recently AISF has proposed a flow-chart that uses clinical, elastographic, US, and laboratory data to distinguish patients at risk for progressive liver disease in order to put them into surveillance programs [23, 24].

The EASL guidelines propose six-monthly surveillance for all patients independently from the etiology who have F4 or F3 fibrosis also estimated through liver stiffness.

The recent AGA guidelines [25], in consideration of the increase in the incidence of NAFLD-related HCC [26] and of the doubts about surveillance programs [27–29], suggest HCC screening only for those patients with compensated cirrhosis or those with decompensated cirrhosis who are listed for liver transplantation. In fact current evidence reports an extremely low incidence on NAFLD at earlier stages of fibrosis (stage 0–2) estimated between 0.03 and 0.6%, and for this reason they do not justify systematic HCC screening [30, 31].

This surveillance is recommended in patients with LC who have a good acoustic window through a bi-annual US evaluation for HCC. On the contrary, in obese patients where US reliability is inadequate [32–34], other imaging modalities (e.g., computed tomography scan or magnetic resonance imaging) are recommended, even though the follow-up interval and their use in association with serum AFP dosage remains to be studied [35]. Further studies are therefore needed to define US surveillance policies to achieve an early diagnosis of HCCs in NASH. The aim of future studies should be the identification of a sub-population of non cirrhotic metabolic patients at increased risk of HCC in whom the surveillance could be cost-effective.

With regard to the echo-patterns in the three periods, the most frequent at the time of diagnosis was the hypo-echoic, and the least frequent was the iso-echoic (7–12%). The mixed pattern gradually decreased from the first to the third group, due to the progressive reduction in the mean size of tumors. This echo-pattern, in fact absent in small nodules, becomes progressively predominant in larger lesions because, as the size of the neoplasm increases, there is an increase in fibrosis, necrosis, steatosis and hemorrhagic phenomena that contribute to the appearance of the inhomogeneous US aspect.

When analyzing the demographic and clinical features of the three groups, we observed that the mean age of HCC patients was progressively increasing from G1 to G3, as already reported in the literature [5, 8, 36–38]. The reasons, especially in patients with HCV etiology, are related to the older age of subjects with HCV-related LC due to the reduced viral circulation as a consequence of a greater control of parenteral transmission routes and the future effects of eradicating HCV infection by direct acting antiviral (DAA) therapies [39]. An interesting fact, however, is that even in the metabolic etiology in G3 there was a higher, though not statistically significant, age compared to G1 and G2.

According to the etiology, we have seen how HCV infection, although it is decreasing, in particular in G3 due to the introduction of DAA therapy, remains the most frequent cause. In contrast to the drop in HCV, our results show that non-viral, especially metabolic, etiology has increased, while HBV remains stable, probably due to migratory flows from geographic areas where vaccination programs are lacking [8].

Our study also finds that metabolic forms are becoming more relevant, both for an absolute increase in HCC on a metabolic basis, as evidenced by the three-fold increase in the number of cases in the last decade compared to the previous one, as well as for a relative increase linked to the reduction of the long-term main cause which has been HCV.

While vaccination programs against HBV, new antiviral therapies, the increased prevalence of post-NASH LC, and bi-annual US surveillance have changed the role of the risk factors, on the other hand they have reduced the severity of HCC presentation at the time of diagnosis. However, there is a significant increase in metabolic etiology in G3 where HCC has a more severe presentation than in the viral etiology, probably due to a delay in diagnosis, related to poor US surveillance and the reliability of the US itself.

## Conclusion

US presentation of HCC over the last years has become less severe, showing a greater frequency of single rather than multiple or diffuse nodules. However, HCCs with metabolic etiology show a more severe staging than viral ones. This raises the need to review the surveillance protocols of patients with metabolic diseases, which will be predominant in the near future, with an integrated serological and elastographic approach to select subjects at higher risk of HCC [40].

## Data Availability

Data are available upon reasonable request to the corresponding author.
